# Expanding the possible: exploring the role for heterodox economics in integrated climate-economy modeling

**DOI:** 10.1007/s43253-023-00098-7

**Published:** 2023-05-17

**Authors:** J. Christopher Proctor

**Affiliations:** 1 Sorbonne University Alliance, University of Technology of Compiègne, Costech laboratory, Compiègne, France; 2 Roma Tre University, Rome, Italy

**Keywords:** Climate change, Heterodox economics, Integrated assessment modeling, Ecological macroeconomics, E11, E12, E14, Q54, Q57

## Abstract

This paper explores the degree to which heterodox economics can contribute to the development and use of climate-economy integrated assessment models. To do so, it introduces the field of integrated assessment modeling, with a focus on the core economic methodology used by various types of models. It then summarizes some of the literature critiquing these models and how they inform policy. The paper then provides an extended classification of ways in which heterodox economics could be applied to climate-economy models and presents a number of storylines, or pathways, which could be created using insights and methods from heterodox schools. The paper concludes with an assessment of the scope for heterodox economics to answer the criticisms of climate-economy models, finding that despite not resolving all issues, the heterodoxy has a substantial role to play.

## Introduction: extreme stories to expand the possibility space

In late February 2020, just days before Europe and the USA joined China and much of Asia in imposing severe mobility restrictions in response to the COVID-19 pandemic, David McCollum and his colleagues published a short Nature Commentary piece calling on climate-economy modelers to “explore extremes” in their modeling (McCollum et al. 2020). Although these models, which attempt to create plausible scenarios of technological, social, economic, and environmental development for the next 80 years, could arguably be described as highly formalized forms of science fiction, the plot-lines investigated by these models are strikingly dull. Where, they ask, are the wars? The geopolitical realignment? The economic panics and shocks? The massive waves of structural economic changes and resulting backlash of social resistance? Where is the space for the wildcards, which are “not even on the radar” (Ibid.)? Their advice was well timed, although their extensive list of specific examples of extremes failed to include the global pandemic which had already quietly begun.

Part of the lack of extreme climate-economy scenarios comes from a failure to pose out-of-the-box research questions to the existing suite of models which have already been built. But part comes directly from the limitations of the models themselves. In their extensive review of the capabilities and limits of the existing integrated assessment modeling literature, Keppo et al. (2021), speak of “exploring the possibility space,” as according to them, “what is not included within the boundaries of the space to be explored cannot be found.” This paper takes the notions of a possibility space for integrated assessment modeling and turns it towards the realm of heterodox economics.

The various schools of thought and research programs that make up heterodox economics typically operate within more open theoretical frameworks and are generally more comfortable with analyzing extreme potentialities than their neoclassical corollaries. From the notion of government-maintained full employment to a vision of prosperity without economic growth, from an understanding of social reproduction and care to a focus on the evolution of institutions and the development of technology, heterodox economics offers a rich range of ideas and tools that could be useful in understanding what policy makers can, and should, do to resolve the climate crisis.

The purpose of this paper is to assess the degree to which the existing suite of heterodox ideas and tools can contribute directly to the development and use of large scale, quantitative, integrated assessment models in order to expand and improve the stories told by these models. To do this, Section. 2 will start with a short review of integrated assessment models and the criticisms levied against them. Section 3 will then procced to a classification of the potential conceptual and methodological contributions heterodox economics could make to understanding the transition to an emission-free global economy, with examples given for how individual concepts could be incorporated. Section 4 will attempt to use the ideas identified as most relevant to create a number of new climate scenarios which would not be readily told with standard climate-economy models. Finally, Sect. 5 will conclude with an assessment of the usefulness of heterodox economics in responding to the limits of integrated assessment modeling.

## Integrated assessment models and their discontents

### Integrated assessment models

Integrated assessment models (IAMs) are a class of quantitative models that combine economic and climatic considerations. These models are built by integrating together a number of separate “modules” which represent different aspects of the economy-climate system. IAMs typically have both an economy module and a climate module which projects emissions and their corresponding levels of warming (Cattan and McIsaac 2021). Simple models stop here, but the most developed IAMs can add many more modules for systems such as energy, technological development, agriculture, land use, and much more. While some simpler IAMs remain fairly abstract, the more complex models can provide an impressive range of projections at a very high resolution, down to the levels of an individual technology adopted in each year (IRENA 2020).

IAMs can be divided into two primary categories according to the research questions they address (IPCC 2022a, 1858). The first, *cost–benefit models*, compare the expected levels of economic damages that will be caused by climate change with the expected costs of mitigating emissions in order to calculate an optimal growth path for the economy. The second, *process-based models*, attempt to replicate the connections between various systems or processes which are relevant for climate mitigation (Ibid.). This involves modeling the linkages between various economic, social, and physical systems to create emission scenarios and climate pathways which represent plausible futures. Because of their orientation towards scenario analysis, process base models can also be called *emission pathway models*.

Prior to the 2015 Paris Agreement, cost–benefit models were frequently used to calculate the optimal level of global emissions, and by extension warming, under various sets of assumptions and model parameters (Nordhaus 1992, 1993). While this research question is still addressed in the post-Paris literature (Glanemann et al. 2020; Hänsel et al. 2020), the clear 2-degree and 1.5-degree limits agreed to by the international community have facilitated a shift in focus from determining the ideal level of warming towards finding pathways to achieving these fixed emission caps.

Emission pathways models have become highly influential in informing national policy and in tracking international progress in mitigating emissions (Süsser et al. 2021). Emissions pathways models are also a core tool of the Intergovernmental Panel on Climate Change (IPCC) reports, with over 3000 scenarios from 191 models submitted for the Sixth Assessment Report on the *Mitigation of Climate Change* (IPCC 2022a, 1882). A representative story of the kinds of features of the scenarios included in the recent IPCC reports can be found in Table 1. As this paper is focused primarily on economic scenario analysis, it will prioritize a discussion of emission pathway models and their economic foundations.

**Table 1:** A representative story of achieving the 1.5- or 2-degree limits according to the scenarios included in recent IPCC reports

Starting immediately, the countries of the world impose a universal emissions tax. This tax will likely need to be at least a few hundreds of dollars per ton of CO_2_ by 2030 to be on track to respect the 1.5-degree limit (Riahi et al. 2022, 360). The tax makes the deployment of a series of decarbonizing technologies—renewable energy generation, nuclear energy, electrification of energy end uses, low-carbon industrial and agricultural techniques, carbon capture, and sequestration—cheaper compared to the more carbon-intensive alternatives. As these technologies are increasingly adopted, they progressively drop in costs, becoming much cheaper by the end of the century (Krey et al. 2019)
In response, greenhouse gas emissions decline sharply, with a global peak reached sometime in the mid-2020s, a global halving of emissions by the early 2030s, and net-zero carbon emissions achieved around 2050 (IPCC 2022b, 17; Riahi et al. 2022, 311). The “net” in net-zero is important however, as the rapid decarbonization is likely not rapid enough, and carbon dioxide will need to be sucked out of the atmosphere to return below the Paris Agreement temperature limits which were overshot sometime after mid-century (Riahi et al. 2022, 311). This drawdown of emissions will likely be both feasible and cost-effective due to large declines in the costs of carbon capture techniques by around 2050. Due to available carbon capture technology, a not-insignificant amount of fossil fuels will remain in use, although coal without direct carbon capture will be nearly totally eliminated (Riahi et al. 2022, 342). While the transition is primarily technological, reductions in demand and more efficient use of energy could play a non-trivial role in the transition, accounting for a potential reduction in emissions of between 5 and 30% by 2050 (IPCC 2022b, 34)
The cost of fully decarbonizing is enormous, with the abatement costs of a ton of carbon reaching tens of thousands of dollars by 2100 (Riahi et al. 2022, 360), implying the need for a global carbon tax as high as $30,000 (IPCC 2018, 152). Over the century, this tax will have the effect of distorting the global economy away from what otherwise would have been its preferred investment path (Riahi et al. 2022, 361). To stay under 1.5 degrees, the global GDP will be roughly 3–4% smaller by mid-century than it otherwise would have been without decarbonization (Riahi et al. 2022, 360). Estimating the effects of the transition on the economy of 2100 is harder, with possibilities ranging from a nearly 10% loss in GDP to a full convergence with the pre-transition growth path (Ibid.). Curiously, despite the significant economic disruption undertaken to constrain emissions, there will be no damages to the global economy from climate change itself during this same period (Riahi et al. 2022, 359)

Author’s elaboration.

The core economics module of each IAM can be designed in a number of different ways. While the results of each model will be determined to a large degree by the specific assumptions and calibrations that go into it, the choice of model structure places key limits on what is possible within the model’s representation of the economic system.

A useful classification of IAMs based on their economic cores is provided by Nikas et al. (2019) who divide IAMs into optimal growth models (corresponding to cost–benefit models above), general equilibrium models, partial equilibrium models, macroeconometric models, and other process-based models. This last “other” category is helpfully expanded upon by Hafner et al. (2020) who add system dynamics models, agent-based models, and stock-flow consistent models.

The most common method for building emission pathway models is a computable general equilibrium (CGE) framework in which the path of the entire economy is optimized, typically at a sectoral level (Matsumoto and Fujimori 2019). Partial-equilibrium models, in which one sector is fully optimized while the rest of the economy is assumed to follow the status quo, are also common, with many partial-equilibrium frameworks focusing on the energy system. These two equilibrium frameworks can be augmented by including various market imperfections and frictions which would prevent the baseline scenario from achieving an optimal state in the short or medium term, but this technique is at the cutting edge of equilibrium climate modeling (Köberle et al. 2021).

Macroeconometric models also include a full representation of the economic system, but unlike CGE models, they are not based on an optimizing function (IPCC 2022a, 1845). Instead, they use real-world data to econometrically calibrate the coefficients of the relationships between the modeled variables, building a web of statistical relationships which can be simulated forward to create scenarios (Lehr and Lutz 2019). This structure allows the overall levels of economic activity to fluctuate within the model based on the demand generated in previous periods, creating more dynamic economic effects than are typically obtained in an equilibrium framework (Mercure et al. 2019). System dynamics, agent-based, and stock flow consistent models, are all associated with heterodox economics and will be described in Sect. 3.

### Their discontents

By their very nature, IAMs are highly interdisciplinary endeavors. This opens them up to criticism from a wide range of fields and perspectives, as their holistic scope touches on dozens of domains across the physical and social sciences. A full accounting of the critiques of IAMs is far beyond the scope of this article, but a summary is necessary to identify the areas where contributions from heterodox economics are most promising. For a more complete review of these critiques, Keppo et al. (2021), Gambhir et al. (2019), and Chapter 3 on *Mitigation Pathways Compatible with Long-term Goals* of the IPCC’s 6th Assessment Report on *Mitigation of Climate Change* (Riahi et al. 2022) are very useful.

A good classification of the types of criticisms of IAMs can be found in the Annex III on *Scenarios and Modelling Method*s from the abovementioned 6th Assessment Report of the IPCC, which divides IAM commentary into four categories (IPCC 2022a, 1862). The first type of criticism is the literature dealing with features and dynamics which are either missing from IAMs or modeled in ways which are perceived to be unfeasible or incredible. The second is a set of concerns around the complexity and comprehensibility of IAMs and their results. The third is a range of critiques about the lack of social, institutional and political elements within IAMs, and fourth is an array of worries about the effects IAMs themselves have on limiting climate policy options.

The first category of literature is the largest, as every parameter, assumption, and model structure is fair game for criticism of being unrealistic or of missing some crucial element. Of particular importance are the assumptions about the development of the costs of particular technologies and the availability of technologies which are currently unproven at large scales (Way et al. 2022). As will be discussed in the next section in greater detail, the macroeconomic assumptions within the models are also grounds for criticism (Asefi-Najafabady et al. 2020; Pollitt and Mercure 2018; Cattan and McIsaac 2021; Riahi et al. 2022, 359).

In terms of complexity, the massive size and scope of most IAMs have raised concerns of a “black box” problem in which results cannot be clearly explained by any identifiable model features (Skea et al. 2021). The issue of complexity is compounded by a lack of transparency of data and code on the part of some models. This opacity means that when considering the results from large sets of models, as is the case in the work of the IPCC, distinctions between results driven by empirics and results driven by theoretical choices can be substantially blurred.

The lack of social and institutional considerations within IAMs may be one of the more surprising features of the models for those not familiar with them, as the scenarios created by the models seamlessly couple dramatic, unprecedented technological transformations with an essentially unchanging global pollical economy (Cherp et al. 2018; Jewell and Cherp 2020). While social dynamics and political change are notoriously hard to quantify and project, the dissonance between the scale of change allowed in some areas of the models and the rigidity of others is striking.

This leads to the final and most fundamental point that IAMs themselves may play a role in closing off doors and “pushing society in certain directions” without proper democratic consent or scientific justification (IPCC 2022a, 1862). Being omitted from IAM scenarios does not mean something has been proven impossible, but omission does have a chilling effect in removing options from the broader policy debate (Gambhir 2019). This critique raises the worry that IAMs are formally cutting off exploration in any direction that requires a structural change to underlying socio-economic systems, and, in so doing, are artificially closing off paths towards better worlds.

## Heterodox economics’ contributions to IAMs: concepts, applications, and methods

For the purpose of this paper, heterodox economics will be considered to be the field of economic research which does not rely on the neoclassical foundations of methodological individualism, optimization, and equilibrium analysis (Arnsperger and Varoufakis 2006; Earle et al. 2016). This includes a wide range of different sub-fields and schools of thought which are both complementary and sometimes contradictory towards each other. Taken together, the broader range of theoretical and methodological starting points contained within heterodox economics provides an inherently expansive view of the possible, allowing for both brighter futures and darker dangers than what is described in the comparatively tranquil world of neoclassical economics. The section will attempt to map some of the potential contributions of heterodox economics in expanding the possibilities allowed within integrated assessment models.

Outlining the contours of heterodox economics, even in the simplest terms, brings a danger of information overload. To better organize the specific ways in which the stock of heterodox knowledge can be used, the paper proposes a classification based on how any given idea could be applied to integrated assessment modeling. At the highest level are *conceptual contributions*, which are key ideas implying deep, structural changes to existing IAMs. A list of conceptual contributions is shown in Table 2. The applicability of these concepts is then expanded upon with an extensive list of indicative examples of how each concept could be applied to a specific topic related to climate-economy modeling in Table 3. Cutting across the broad concepts and the narrow examples are methodological techniques which can augment or replace the equilibrium modeling which is currently standard IAMs. These methodological contributions are detailed in Table 4.

**Table 2:** Potential conceptual contributions to IAM modeling from the various heterodox schools of economic thought

School of thought	Concept		
Post-Keynesian	Effective demand	Fundamental uncertainty	Endogenous money
Evolutionary	Path dependency^a^	Heterogenous agents	Creative destruction
Feminist	Wellbeing	Social reproduction	Cost-disease
Ecological	Bio-physical embeddedness	Social metabolism	
Marxian	Power	Exploitation and class	
Institutional	Institutional embeddedness	Legal institutionalism	
Cooperative	Economic democracy		
Behavioral	Prospect theory		

Author’s elaboration.

^a^Path dependency is also closely associated with Post-Keynesian economics.

**Table 3:** Descriptions of heterodox concepts and potential applications to integrated assessment modeling

Concept	Short description of the concept	Example of potential applications to integrated assessment modeling
Effective demand	Economies are typically constrained by aggregate demand and not supply factors	• Introducing positive feedback loops between green investments and growth • Linking growth dynamics to changes in distribution between wages and profits • Differentiating between economic effects of carbon pricing versus industrial policy
Fundamental uncertainty	Probabilities for many key events are unquantifiable	• Incorporating extreme climate damage functions • Considering continuous ranges of inputs rather than discrete scenarios
Endogenous money	Money is created cyclically within the financial system	• Endogenizing boom and bust driven by changes in decarbonization policy • Directly linking sustainable finance and technology deployment
Path dependency	Events in one period shape the possibilities in the next	• Making the availability of various technology and policy options dependent on earlier model outcomes • Conceptualizing a range of intermediary mid-way points for scenarios to transverse
Heterogenous agents	Differences between actors are essential for understanding economic dynamics	• Differentiating between green and brown firms based on emission intensity • Modeling changes in behavior leading to demand reductions • Creating different decision-making heuristics for countries based on material interest
Creative destruction	Capitalism reconfigures itself, destroying old structures while raising new ones	• Incorporating the innovative role of the state via R&D policy variables • Connecting technology costs to a detailed knowledge economy module
Wellbeing	Policy should target improving a broad concept of human wellbeing	• Introducing composite wellbeing indicators to IAMs • Integrating wellbeing as a feedback or limit on other variables
Social reproduction	The continuation of social systems requires non-market labor	• Explicitly tracking rates of non-market care work in rapid transition scenarios • Breaking down households in climate damage functions to model gendered effects
Cost-disease	Personal services become relatively more expensive over time	• Projecting large shifts in labor use towards in-person services in 2100 scenarios • Estimating the impact of cost disease on long-term technology cost projections
Bio-physical embeddedness	Economies are constrained sub-systems of larger biological and physical systems	• Introducing hard energy, material, and land use limits which curtail growth • Incorporating measurements of non-climate planetary boundaries into climate scenarios • Building non-climate damage functions for failures of other ecological systems, like biodiversity
Social metabolism	Society is based on flows of energy and materials between nature and the economy	• Modeling changes in sectoral energy and material use economy-wide • Explicitly modeling the physical waste implied by economic activities • Experimenting with negative growth scenarios
Power	Power imbalances are a defining feature of capitalist economies	• Creating explicit politics sub-modules to endogenize policy ambition • Allowing for sudden shifts in economic structure • Modeling relative sectoral power, with tipping points allowing policy changes
Exploitation and class	Capitalists create profit by extracting value from workers	• Explicitly modeling sectoral profit rates • Estimating the distribution of climate damages between capital and labor
Institutional embeddedness	Markets are intrinsically intertwined with social institutions	• Modeling institutional collapse with tipping points triggered by low levels of wellbeing • Differentiating countries and regions by political-economic system • Calibrating models to historic cases of rapid institutional transition
Legal institutionalism	Market economies are constituted by laws and legal frameworks	• Modeling asset stranding under different property rights regimes • Including specific processes laid out in international agreements in a policy module
Economic democracy	Economies can be managed democratically at a micro and macro level	• Differentiating firms based on decision making and ownership structures • Simulating the de-concentration of key sectors like energy and agriculture
Prospect theory	Losses are relatively more painful than gains are pleasurable	• Including wider policy options for managing losses from stranded assets • Increasing the focus on modeling subsidies compared to emission taxes

Author’s elaboration.

**Table 4:** Methodologies associated with heterodox economics relevant for integrated assessment modeling

Method type	Method	Short description	Examples
Modeling frameworks	Macroeconometric	Simulation models based on large sets of econometric correlations	Pollitt and Mercure (2018); Mercure et al. (2018)
	System dynamics	Modeling framework based on stock and flows and incorporating feedbacks, delays and non-linearities	Meadows et al. (1972); Capellán-Pérez et al. (2020); Dixson-Declève et al. (2022)
	Stock flow consistent	Models built on strict accounting frameworks ensuring consistency between financial stocks and flows	Monasterolo and Raberto (2018); Dafermos et al. (2017); Jackson and Victor (2020)
	Agent-based	Models created by simulating the interactions of autonomous heterogenous agents	Czupryna et al. (2020); Lamperti et al. (2018); Lamperti and Roventini (2022)
Supporting methods	Input output analysis	A form of data analysis which shows the physical and financial flows between sectors in the process of production	Nieto et al. (2020; 2023)
	Participatory modeling	A practice of systematically involving stakeholders at various points in the process of creating and using models	Jones et al. (2009); Dolter (2021)
	Contextualized modeling	A concept applied to the use of models which attempts to contextualize their contributions and acknowledge their limits	Doukas and Nikas (2020); Gambhir (2019)
	Multi-model analysis	The practice of using suites of different models to gain deeper insights about a research question	Doukas et al. (2018); Sognnaes et al. (2021)

Author’s elaboration, modeling frameworks adapted from Hafner et al. (2020)

The concepts presented in Table 2 cover some of the “big ideas” from heterodox economics. These ideas are more than just topical interests, but instead are different ways of looking at the economy. Each highlights a feature of the economic system which, if emphasized, leads to very different lines of research and analysis. Each concept is further subdivided by the school of economic thought they are most closely associated with, with the specific schools of thought adapted from Fischer et al. (2018). While invoking schools of thought is always fraught with the danger of over-simplification, it is hoped that this further subdivision will help clarify the ideas in question. While the concepts cover a wide range, they were selected for their potential applicability to climate-economy modeling and are not intended to fully cover the contributions of heterodox economics more generally. The objective of identifying these concepts is to allow for clearer discussion of the sorts of stories which could be readily built using the building blocks provided by heterodox economics, a task which will be expanded upon in Sect. 4.

To provide context for this list of concepts, a short description of each and examples of how each could be applied to integrated assessment modeling is provided in Table 3. These examples are indicative of the scope of specific interventions which are open to heterodox modelers, and range from those which are straightforwardly implementable (i.e., incorporating extreme climate damage functions or introducing composite wellbeing indicators within IAMs) to those which would require the creation of entirely new climate-economy models. Most of the proposals are compatible with one another, and taken together, they represent a path towards formally synthesizing insights from various schools of heterodox economics.

Finally, Table 4 outlines various research methods which are directly applicable to creating and using emission pathway models. The methodological contributions are divided by the nature of their potential use, with primary modeling techniques being separated from supporting methods. Short descriptions and references to examples are given for each. Compared to the list of concepts, the range of methods is narrower, with priority given to the techniques which are already in use. In particular, the list of modeling frameworks draws directly from the model types reviewed by Hafner et al. (2020).

The concepts, applications, and methods presented in this section will be illustrated further in the following section, where they will be used to tell the stories of various potential climate futures.

## Telling new stories with heterodox concepts

To enable easier comparison across models, the IAM community has collaborated to create a set of common narratives which depict potential pathways for the world’s political-economic development over the coming century. These stories, called the Shared Socioeconomic Pathways^1^ (SSPs) provide specific background inputs for population, urbanization, education, and GDP growth for five different global scenarios and are used widely in the modeling community (Riahi et al. 2017; O’Neill et al. 2017). These sets of inputs are each categorized according to the degree to which they make either mitigating or adapting to climate change easier or more difficult. This creates a matrix of scenarios detailing the various options of overall low challenges, overall high challenges, low challenges for mitigation but difficult adaptation, and finally high challenges for mitigation but low obstacles to adaptation (Kriegler et al. 2012; O’Neill et al. 2014).

**Fig. 1:**
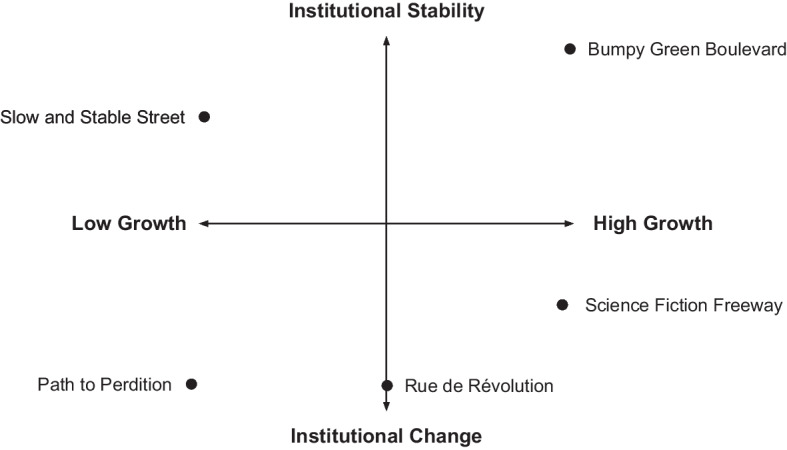
Climate-economy pathways organized by levels of growth and institutional change (author’s elaboration)

As an example of how heterodox concepts can be put to use, this section will now propose a similar framework of socioeconomic background scenarios which could serve as starting points for modeling projects. Rather than organizing the scenarios along the axes of mitigation and adaptation, we propose a categorization on the basis of the levels of economic growth and institutional change in each storyline. This classification allows us to more systematically explore fundamental changes to both political-economic structures and the underlying growth path of the economy, two features which are not readily treated in the existing IAM literature. Figure 1 depicts the five scenarios detailed in this section, arranged approximately along the beforementioned axes.

Building off of the heterodox concepts listed in Sect. 3, each scenario also highlights a number of concepts which would be particularly important for modeling the pathway. While a large number of the concepts are fully compatible with each other and should be considered in any scenario built on heterodox foundations, the concepts identified as particularly relevant are those which would likely need to be explicitly modeled in a formal representation of each particular scenario. Each scenario will also be linked with examples of methods which could be readily applied to it, with preferences given to references to existing work where possible. The remainder of the paper will detail the pathways in Fig. 1, starting from a narrative description of each scenario.

### Bumpy Green Boulevard

*Particularly relevant concepts: effective demand, bio-physical embeddedness*

Countries around the world embrace the environmental transition as an opportunity to fight climate change while boosting sluggish post COVID-19 economies. This ‘green growth’ strategy initially pays off, as green investments spark an economic boom which keeps workers employed and demand strong. Unfortunately, as the transition is truly getting off the ground, the global economy starts to run up against hard limits to energy, material and land availability. These limits slow not only the transition, but economic activity more generally, as countries struggle against each other to find enough inputs to maintain growth.

In this story, the concepts of effective demand and biophysical embeddedness are emphasized to tell a cautionary tale about potential non-economic limits to the speed of the transition. In a standard neoclassical IAMs this story would look quite different. Firstly, the large-scale green investment campaigns would not be met with a spike in growth due to increased aggregate demand, but would rather be fully crowded out by reductions in investments elsewhere in the economy. Furthermore, as the green investments are only being made because of a policy intervention, they would be assumed to be less productive than whatever was crowded out, meaning the long-term growth potential of the economy would be somewhat lower. Secondly, while standard IAMs can and do track the amount of energy, materials and land associated with various pathways, they do not typically use these features to impose binding limits on either sectoral or economy-wide growth.

In terms of actually modeling this story, a very similar scenario has already been created using the MEDEAS World model (Capellán-Pérez et al. 2020; Nieto et al. 2020). Within MEDEAS, the economy is represented as demand-led, with an input–output framework providing insights about how demand is distributed across the various sectors of the economy. As a system dynamics model, MEDEAS also includes hard energy availability feedbacks which prevent desired demand from being fully fulfilled unless the energy submodule is able to generate a sufficient amount of energy. In similar scenarios in MEDEAS to the story presented above, deployment of new renewable energy infrastructures is unable to keep up with the quick pace of “green growth,” leading to a relative scarcity of fossil fuels and unintentional near-zero growth rates (Nieto et al. 2020).

Comparable demand-led growth effects also exist in macroeconometric models, although so far without an emphasis on energy limits (Pollitt and Mercure 2018; Mercure et al. 2019). Along with full integration into heterodox IAMs, the concepts of effective demand and bio-physical limits can also be applied in ad hoc ways to equilibrium models by testing their results under significantly higher growth paths and by conducting feasibility analyses of the levels of material and energy inputs required at various points in a given scenario.

### Slow and stable street

*Particularly relevant concepts: social metabolism, wellbeing, and endogenous money*

After a nasty set of energy crises in the early 2020s, the countries of the European Union decide to stop pursuing economic growth as a policy goal and instead target a broader index of wellbeing. While the shift in goals is certainly radical, it leaves the fundamental structure of Europe’s economy largely in place. This leads to some financial difficulties, particularly concerning public debts. To overcome these challenges, international financial institutions are forced to adapt, creating a more flexible financial regime for both Global North and Global South alike.

By combining a reduction in the rate of social metabolism with a focus on wellbeing, this story describes a path characterized more by a slowing of environmental pressures than a mad dash of technological deployment. The scenario is enhanced by a detailed examination of what this slowing means for the financial system and public finances, each of which could become problematic as credit endogenously contracts alongside the slowing economy. In principle, the slowing of growth could be explored with existing IAMs by simply inputting a desired growth path which converges near zero. This, however, would miss the complications a lack of growth would cause for the financial system and public finances, features which are not readily included within standard equilibrium IAMs.

Versions of this story have been a key starting point for a number of heterodox modeling exercises, with Jackson and Victor (2020) and Dafermos et al. (2017) doing so in a stock flow consistent modeling framework and D’Alessandro et al. (2020) with a system dynamics model. Key insights to come out of this work include the macroeconomic feasibility of such a path and a warning about the role public debt in particular may play as economic activity plateaus or contracts. The possibility of integrating wellbeing as a target within modeling exercise has also been shown, for example in Jackson and Victor (2020) who track a “sustainable prosperity index” and in the “average wellbeing index” of the Earth4All system dynamics model (Dixson-Declève et al. 2022). The concepts of social metabolism and wellbeing are also good starting points to further analyze the question of global justice during the ecological transition (Rubiano Rivadeneira and Carton 2022).

### Science fiction freeway

*Particularly relevant concepts: creative destruction, path dependency, and heterogenous agents*

Recognizing the difficulties of honoring the 1.5-degree limit with existing technology, a core group of countries enter into an International Innovation Club. Membership in the club requires large public funding of green research and development and mandates that all results are shared openly. By coordinating and expanding the world’s knowledge creation, the Club manages to shock the world, introducing new techniques and technology which dramatically reshape the direction of the transition. Old problems are replaced with new however, as the solutions to the climate crisis put pressures on other ecological systems, and new inequalities arise between member and non-member countries.

Current IAMs typically rely on specific sets of individual technologies for their projections. This makes sense in the near term: it is fairly unlikely that something no one has ever heard of will be a major player in the transition over the next decade. In the longer term it is less clear, as if history is any guide, the types and levels of technology in 2100 will likely be quite different than today. Capitalism has a powerful capacity to create, especially under specific institutional and legal conditions which allow for coordination and cooperation. Combined with the notion of deep path dependency, a concerted effort to alter our technological path could potentially take us places that are currently difficult to imagine.

In terms of modeling, what is needed to better tell this story is a more explicit representation of technological change. This should certainly include possibilities for the refinement of existing technologies (Way et al. 2022), but ideally would also include space for new tools to emerge based on the needs displayed in a given scenario. A promising path forward could be with agent-based models, which allow the differentiation between innovative and static actors and the comparison of various knowledge-sharing regimes. Agent-based models have already been used extensively to study innovation and technology, and have recently been combined with climate modules to create IAMs (Dosi et al. 2010; Lamperti et al. 2018, 2020). A further step could be to build modules depicting state-led innovation programs to be integrated as policy options within larger IAMs. The models could also explore the implications of various technological paths for development and international wealth distribution.

### Path to perdition

*Particularly relevant concepts: Institutional embeddedness, social reproduction, and fundamental uncertainty*

The horrors of climate change, which had always seemed distant, arrive with a fury in the 2030s. While the direct damage from not-so-natural disasters are substantial, the more serious issues come from the gradual breakdown of political and social institutions, as democracies become less democratic and the rule of law routinely fails. Things which were once taken for granted, like public educational and health systems, become fragile or non-existent, shifting burdens once shouldered by the state on to families and local communities. The old distinctions between developing and developed countries fade, as new divisions arise between failed and functioning societies. While the global economy continues to muddle through the chaos, it is a shadow of it’s early 21^st^ century peak.

Most emission pathway IAMs are designed to better understand how society can limit global warming. This makes them somewhat utopian, as in both their construction and the bulk of their scenarios, they are exploring some of the brightest possible futures. More work could be done in exploring the other side of the bell curve, in trying to understand what could happen if we fail. Here, the concepts of institutional embeddedness and social reproduction could be very useful for understanding how things turn from bad to worse should the social and institutional pillars holding up market economies falter. The recognition of the often-overlooked prerequisites of our economic systems, along with an honest appraisal of the fundamental limits to our abilities to know what is around the corner, allow for the telling of a much more sober story than what is implied by measuring the effects of shocks within an equilibrium framework.

Heterodox scholars have already extensively criticized existing efforts to estimate potential climate damages (Keen 2020; Woillez et al.^2020^; Asefi-Najafabady et al. 2020). A new challenge would be to construct an alternative modeling framework that examines not just climate changes’ effect on productivity or growth in a given year, but its cumulative effect on political, institutional, and social systems. Of particular importance would be recognizing the impact of climate change on activities like care work which often take place outside of markets but are essential for supporting market economies (Pearse 2017; Eastin 2018). This line of modeling would also allow space for international comparisons of institutional resilience.

By identifying possible tipping points and subsequent chain reactions, this line of modeling could provide a more complex and detailed pictures of what a warming world could look like. This would be useful not only as a warning in the here and now, but also as an improvement to the internal logic of the IAMs themselves, as the addition of social and institutional loops would force actors within the modeled world to face clear and severe consequences if they decide to shirk on mitigation.

### Rue de Révolution

*Particularly relevant concepts: power and economic democracy*

What starts as a protest movement grows into something bigger, as wide swaths of civil society come together to demand something different. Starting from the periphery of the global economy and spreading country by country towards the core, the result is an economy transformed from below, with massive global corporations replaced by local and regional cooperatives, and the rule of the free market replaced with democratic planning. The economy is transformed, but ecological challenges remain as the leaders of the new system struggle to balance environmental concerns with the material interests of the people who they serve.

Standard climate-economy models explore a world in which the technology that underlays our economy changes dramatically, but the fundamental structure of the economy, and the broader society it supports remain essentially the same. Not only is this not the only possibility, but it also seems a fairly unlikely one. Social upheaval and revolution are a core part of the story of the first Industrial Revolution, and it remains entirely possible that something comparable could accompany an ecological transition. Power is a clear conceptual building block for analyzing societal transformations, and when combined with the idea of economic democracy, one can start to see the broad shape of a very different economic system.

Modeling this new system, the transition that creates it, and its interaction with the core environmental and technical challenges of decarbonization, is a tall task, which to the author’s knowledge has not been fully embraced by any large-scale integrated modeling projects. At the macro level, system dynamics modeling could be flexible enough to accommodate entirely different political-economic systems within the same model, with social tipping points endogenously pushing countries from one system to another, while at the micro level, an agent-based representation could simulate the social processes which lead to system changes. Perhaps a concrete way forward would be through participatory modeling, in which non-modelers build detailed descriptions of existing and potential systems that modelers then attempt to formalize. A set of such formalized alternative economic systems then could serve as a kind of post-capitalist Shared Socioeconomic Pathways, providing common end points to reconstruct within the various heterodox IAMs.

## Conclusions: the role of heterodox economics in expanding the possible

The previous section shows that the concepts and methods of heterodox economics could be used to tell different stories than those which are currently told by neoclassical integrated assessment models. The final task then is to assess whether telling these additional stories would actually be an improvement to the landscape of climate-economy modeling. To approach this assessment, it is helpful to recall the typology of common criticisms against IAMs presented in the 6th IPCC Assessment Report and summarized in Sect. 2, which included criticisms of IAMs for (1) having missing or incorrect assumptions, (2) being overly complicated and unclear, (3) lacking social and institutional consideration, and (4) for being used to close doors to unmodeled possibilities.

On the first count, of adding and substituting assumptions, heterodox economics has much to contribute, as elaborated in many of the applications shown in Table 3. In terms of substituting existing assumptions, one concept that stands out is effective demand, which can literally turn the economics of IAMs on its head by flipping losses from mitigation into gains. In terms of new assumptions to be added, the bio-physical embeddedness of the economy is a clear priority, as current models lack even damages from climate change, let alone interactions and limits coming from other physical and ecological systems.

For the criticism of the opacity of IAMs, the contribution of heterodox economics is less clear. If anything, the long list of new complexities suggested by a heterodox analysis has the effect of increasing, not decreasing, how complicated new IAMs would need to be. Especially when attempting to formalize multiple concepts within the same model structure, there is a real danger of losing track of the effect of any given assumption to the larger picture. Similarly, when trying to model large, systemic shifts in one part of a model there is a possibility that the nuances of other parts of the model will be overwhelmed. The additional complexity called for by heterodox modeling also brings with it serious technical challenges for actually building and running quantitative models which can convincingly represent real world systems. This balance between realism and complexity is a key challenge for potential heterodox IAMs.

For the criticisms that IAMs need stronger social and institutional contextualization heterodox economics again has a clear contribution to make, with a number of entire schools of thought dedicated to analyzing the broader societal context within which economies operate.

But it is in the critique that IAMs should be used in ways that do not artificially close off viable policy options, in which perhaps the strongest argument for involving heterodox economics in integrated assessment modeling emerges. No economic theory or methodology can describe every reality or every possibility. As such, a plurality of perspectives and tools are needed to have a true sense of what the future may have in store for us. The concepts and techniques used within heterodox economics can be used to describe and explore scenarios which are currently impossible in neoclassical climate-economy models yet remain entirely plausible in the real world. This suggests a strong and persistent role for heterodox ideas and heterodox scholars in building, adapting, and improving emission pathway scenarios and the models which create them.

Not every story can or should be told through formal, quantitative models. But more stories, and more stories which are considered extreme, must be told if humanity hopes to achieve its climate goals as laid out in the 2015 Paris Agreement.

## Data Availability

The paper presents no data.

## Footnotes

1 For reference, the names given to the five shared socioeconomic pathways (SSPs) are (1) Sustainability — Taking the Green Road; (2) Middle of the Road; (3) Regional Rivalry — A Rocky Road; (4) Inequality — A Road Divided; and (5) Fossil-fueled Development — Taking the Highway.
